# Toxicity Assessment of Chinese Herbal Medicine* Cynomorium songaricum* Rupr

**DOI:** 10.1155/2019/9819643

**Published:** 2019-03-04

**Authors:** Fenfen Wei, Qinghua He, Wenjuan Wang, Dong Pei, Bo Zhang

**Affiliations:** ^1^Beijing Key Laboratory of Bioactive Substances and Functional Food, Beijing Union University, Beijing, China; ^2^Lanzhou Institute of Chemical Physics, Chinese Academy of Sciences, Gansu Province, China

## Abstract

*Cynomorium songaricum* Rupr, known as Suo Yang, is most commonly used to treat fatigue, protect the liver, and invigorate kidneys in Northwest China. Given the wide medicinal utilisation and lack of safety evaluation, this work evaluated the acute toxicity, genetic toxicity, and 90-day repeated oral toxicity of Suo Yang. Twenty Kunming mice were orally given Suo Yang once and observed for 14 days in the acute toxicity test. The genetic toxicity of Suo Yang was tested using in vivo and vitro assays (bacterial reverse mutation test, mouse bone marrow micronucleus assay, and spermatocyte chromosomal aberration assay). In the 90-day repeated oral toxicity study, 80 SD rats were randomly divided into 4 groups and then orally given Suo Yang at different concentrations (1.04, 2.08 or 4.16 g/kg), while the control group was given sterile water. In the acute toxicity test, no abnormal behaviour or mortality was found in mice. The results suggest that the maximum tolerable dose of Suo Yang is greater than 15 g/kg. In the genotoxicity studies, no revertant colonies were produced in vitro. In the in vivo assays, no increased frequencies of micronuclei or structural abnormalities of spermatocyte chromosomes were found. In the 90-day repeated oral toxicity study, no significant toxicological manifestations were observed in haematological parameters or clinical and pathological examinations. In summary, these results suggest that Suo Yang at the given doses does not cause adverse effects in animals. Thus, Suo Yang can reasonably be considered a safe herbal medicine.

## 1. Introduction

*Cynomorium songaricum* Rupr, known as Suo Yang, belongs to the genus Cynomorium, the sole genus within the* Cynomoriaceae* family. Suo Yang plants are holoparasitic and have no leaves [1, 2], so they cannot produce energy by themselves. They usually parasitise the roots of other plants, such as those of the Nitrariaceae family in China [3]. In Northwest China, Suo Yang has been used as food and medicine for hundreds of years [4]. This herbal medicine is most commonly used to treat fatigue, protect the liver, and invigorate the kidney [5]. Suo Yang was mentioned by Tao Zongyi (1329-approximately 1410) in his* Bencao Yanyi Buyi* (*Supplement and Expansion of Materia Medica, 1347*). The values of Suo Yang were similarly described in the famous Chinese pharmacist Li ShiZhen's work* Compendium of Materia Medica *(1578) and other ancient Chinese medicine works (such as* Danxi Xinfa, Bencao Yuangshi*, etc.) [6].

The major constituents isolated from Suo Yang include flavonoids, triterpenes, organic acids, steroidal compounds, and polysaccharides [7]. In recent decades, much attention has been paid to Suo Yang due to its important physiological properties associated with health benefits. Jiang et al. analysed two new phenolic compounds isolated from the stems of Suo Yang and tested their biological activity [6]. Another study showed that flavonoid extracts (containing rutin, catechin, and isoquercetin) isolated from Suo Yang can increase the activity of enzymes that scavenge reactive oxygen species (ROS) and enhance exercise performance in rats [7]. Yang et al. investigated the effects of Suo Yang on sperm parameters and glial cell line–derived neurotrophic factor (GDNF) expression in rat testes [8]. Their results suggest that Suo Yang may improve male fertility by enhancing spermatogenesis and GDNF expression. Liu et al. adopted a pharmacological approach using Drosophila to study the mechanisms underlying the role of Suo Yang in antisenescence [9]. The results showed the possible clinical utility of Suo Yang for slowing the ageing process.

Although Suo Yang is the most common Chinese herbal medicine, its limits in current therapy and adverse effects on conventional drugs remain unclear [10]. In recent years, with rapid global economic integration, China's pharmaceutical industry has moved strongly into the international medicine market [11]. Thus, a study of Suo Yang's toxic effects and determination of a safe dose are urgently needed [2]. To understand the toxic effects of Suo Yang and further promote its application, we performed this study in which single-dose acute toxicity of Suo Yang was assessed in mice. In addition, the bacterial reverse mutation test (Ames assay), mouse bone marrow micronucleus test, and mouse spermatocyte chromosomal aberrations were used to evaluate the genotoxicity of Suo Yang. Furthermore, a 90-day repeated oral treatment test in rats was used to examine the subchronic toxicity of Suo Yang.

## 2. Methods

### 2.1. Test Substances

Suo Yang (certificate no. SY20151207) was supplied by Lanzhou Institute of Chemical Physics, Chinese Academy of Sciences (Lanzhou, China). The samples were ground to powder from the roots of Suo Yang and filtered through 80 mesh screens. The composition of Suo Yang was analysed and found to be sugar (26.9%), starch (14.7%), protein (8.69%), ash (7.8%), flavonoids (5.64%), polysaccharide (3.45%), sucrose (3.09%), fat (1.6%), catechins (0.031%), and water.

### 2.2. Chemicals and Reagents

The following chemicals were purchased from Sigma-Aldrich (Saint Louis, MO, USA): 2-aminoanthracene, 1,8-dihydroxyanthraquinone, Endoxan, colchicine, sodium azide, and dimethyl sulfoxide (DMSO). The S9 mix was obtained from Molecular Toxicology, Inc. (Boone, NC, USA). Giemsa stain was purchased from Nanjing Jiancheng Bioengineering Institute (Nanjing, China). Methanol was obtained from Beijing Dingguo Changsheng Biotechnology Co., Ltd. (Beijing, China).

### 2.3. Animals

The experimental protocol was reviewed and approved by the Ethics Committee of Beijing Union University for the use of laboratory animals. In this study, two kinds of animals were used. Kunming mice were used for the acute toxicity and genetic experiments, and Sprague–Dawley (SD) rats were used for the 90-day repeated oral toxicity study. All animals were specific pathogen-free (SPF; certificate no. SCXK2012-0001), provided by Beijing Vital River Laboratory Animal Technology Co., Ltd. (Beijing, China), and were housed in an SPF animal room (SYXK2012-0031). The animals were kept under controlled conditions (temperature, 22 ± 2°C; humidity, 50-60%) with a 12 h light–dark cycle and were allowed free access to feed and water. The feed was provided by Beijing HFK Bioscience Co., Ltd. (certificate no. SCXK2014-0008).

### 2.4. Acute Toxicity Experiment

A total of 20 (10 males and 10 females) mice (18 g ~ 20 g) were used for this test. Suo Yang powder was dissolved in sterile water. The mice were given the powder by oral gavage as a single dose of 18 g/kg in a volume of 40 ml/kg (equivalent to 432 times the clinical human dosage). Over 14 days, they were monitored for mortality, body weight, and clinical signs. At the end of this experiment, animals were euthanised by isoflurane inhalation and then decapitated.

### 2.5. Genetic Toxicity Study

#### 2.5.1. Bacterial Reverse Mutation Test

To analyse the ability of Suo Yang to induce mutations, a bacterial reverse mutation assay was performed. According to the recommended methods,* Salmonella typhimurium* (TA97, TA98, TA100, TA102, and TA1535) was selected for this assay. All strains were purchased from Molecular Toxicology Inc. (Boone, NC, USA). To determine the dose range, preliminary tests were conducted with a high concentration, 5000 *μ*g/plate, and then the plates were sterilised at 121°C for 20 min. Then, plates were placed in a sterile environment for 24 h at 37°C. On the basis of these preliminary tests, Suo Yang powder was diluted with sterile water at 5 different concentrations, including 8, 40, 200, 1000, and 5000 *μ*g/plate, with and without exogenous metabolic activation (S9 mix). Before water and DMSO were used, they were sterilised at 121°C for 20 min. With S9 metabolic activation, a positive control, 2-aminoanthracene, was used at a concentration of 10 *μ*g/plate for strains TA97, TA98, and TA100. For the bacterial strain TA102, 1,8-dihydroxyanthraquinone was used at a concentration of 50 *μ*g/plate, and cyclophosphamide was used for TA1535 at a concentration of 200 *μ*g/plate. Without S9 metabolic activation, dimethylaminobenzene diazosulfonate sodium was used as a positive control at a concentration of 10 *μ*g/plate for strains TA97, TA98, and TA102. For bacterial strains TA100 and TA1535, sodium azide was used at a concentration of 1.5 *μ*g/plate. Subsequently, 0.1 ml bacterial culture, 0.1 ml sample solution, or 0.5 ml S9 (when activation was needed) was poured into glucose agar plates and incubated at 37 ± 1°C for 48 h. Finally, the numbers of revertant colonies were calculated. To ensure the reliability of the results, the test was repeated 3 times under the same conditions. The results were considered positive if the number of colonies in the test group was more than 2 times higher than that of the spontaneous regression (untreated control) group.

#### 2.5.2. Mouse Bone Marrow Micronucleus Assay

For the erythrocyte micronucleus assay, 50 mice (25 males and 25 females; 25~30 g) were randomly separated into 5 groups (10/sex/group). Suo Yang powder was diluted with sterile water at 3 different concentrations (2.25, 4.50, and 9.00 g/kg). Negative and positive control groups were given vehicle (20 ml/kg) and cyclophosphamide (40 mg/kg), respectively. The respective treatments were administered once a day for 2 days. At 6 h after the last administration, animals were euthanised using isoflurane inhalation and then decapitated. Their sternums were excised to obtain bone marrow cells. The obtained cells were suspended in foetal bovine serum (FBS), smeared on slide glasses, dried, and fixed with methanol for 5 min at room temperature. Finally, these cells were stained with Giemsa, and two slides were prepared for each animal. The micronucleus rate was counted among 1000 polychromatic erythrocytes (PCEs) per animal. Slides were examined under a Nikon biological microscope at a magnification greater than 400× to observe micronuclei of PCEs. The results were evaluated statistically by analysis of variance (ANOVA). Micronucleus ratios that were significant at one or more doses were reasonably considered a positive result.

#### 2.5.3. Spermatocyte Chromosomal Aberration Assay in Mice

For this assay, 25 mice were randomly divided into 5 groups (5/group): solvent control group (20 ml/kg), positive control group with cyclophosphamide (40 mg/kg), and three dose groups (2.25, 4.50, 9.00 g/kg). The solvent group and dose group were orally given sterile water or Suo Yang in a volume of 20 ml/kg for 5 days, respectively. At the same time, the positive control group was given Endoxan by injection once a day. On the 14th day, all animals were injected with colchicine (5 mg/kg) in a volume of 10 ml/kg. After 5 h, the mice were euthanised by isoflurane inhalation and then decapitated, and the epididymis was excised and shredded in 2 ml saline. Then, the samples were mixed with trisodium citrate (w/v, 1%), centrifuged and stained with Giemsa. These samples were analysed under a Nikon biological microscope. Nonoverlapping (n = 1000) spermatocytes were observed for each mouse and included fragments, microchromosomes, and translocations. The spermatocyte malformation rate (%) was calculated.

### 2.6. 90-Day Repeated Oral Toxicity Study

For this study, 80 SD rats (40 males and 40 females) were randomly divided into four groups: group 1, vehicle control group; group 2, 1.04 g/kg; group 3, 2.08 g/kg; and group 4, 4.16 g/kg (equivalent to 25, 50, and 100 times the clinical human dosage, respectively). Animals were treated daily at 8 a.m. with gastric infusions in a volume of 10 ml/kg for 90 days. During the administration period, mortality, signs of gross toxicity, and general behaviour of all animals were monitored. During the experimental periods, to avoid drug accumulation and analyse the causes of death, dead rats were immediately collected for anatomical analysis [12]. Rats were sacrificed after blood was collected and were opened surgically for evaluation of possible pathological changes.

#### 2.6.1. General Conditions, Body Weight, and Food Utilisation Rate

During the 90-day administration period, each animal was monitored for mortality, body weight, food utilisation rate, and clinical signs. Individual animal body weight and food consumption were measured once a week. Final body weight was recorded prior to the scheduled necropsy. The mean body weight and mean food utilisation rate were calculated for the corresponding intervals.

#### 2.6.2. Haematological and Clinical Biochemical Analysis

Haematological parameters were determined with an automatic blood analyser and included red blood cell count (RBC), haemoglobin concentration (HB), total white blood cell count (WBC), granular leukocyte count (GLC), and lymph leukocyte count (LLC). Clinical biochemistry parameters were detected using an automatic chemical analyser and included alanine aminotransferase (ALT), aspartate aminotransferase (AST), total protein (TP), albumin (ALB), blood urea nitrogen (BUN), creatinine (CREA), blood glucose (GLU), cholesterol (TC), triglyceride (TG), potassium, and sodium.

#### 2.6.3. Organ Weight, Relative Organ Weight, and Histopathological Analysis

After blood was collected, rats were sacrificed and opened surgically for evaluation of possible pathological changes. The liver, spleen, lungs, kidneys and testes were all weighed to calculate the relative weight of organs according to the formula organ coefficient = [(organ weight/body weight) × 100%] [13]. The liver and kidneys were fixed in 10% formalin buffer for further research. Dehydration, embedding, sectioning, dewaxing, and staining with haematoxylin-eosin were performed only on the high dose group initially [14]. Then, these stained organs were assessed under an optical microscope at 400× magnification.

### 2.7. Statistical Analysis

The data were analysed using SPSS 11 software. All data are expressed as the means ± standard deviations. First, the experimental data were examined for variance homogeneity. When the test results indicated no significant deviations from a homogeneous variance, one-way ANOVA was conducted. When significant deviations were noticed, a multiple comparison test (Dunnett's test) was performed to determine which groups were significantly different from each other. If significant deviations from homogeneous variance were observed, a nonparametric test was performed. The level of significance used was* P*<0.05 or 0.01.

## 3. Results

### 3.1. Acute Toxicity Experiment

The results of the acute toxicity assay are shown in Table 1. Mice were orally given a maximum concentration and maximum volume (equivalent to 432 times the clinical human dosage). During the 14 days of observation, the animals appeared normal, showing no signs of poisoning and no mortality. Exterior appearances and behaviours were normal, and no gross lesions were found on internal organs during autopsy, indicating that the maximum tolerable dose of Suo Yang in mice is greater than 15 g/kg. According to toxicological standards and the experimental results, Suo Yang can reasonably be considered nontoxic.

### 3.2. Genetic Toxicity Study

#### 3.2.1. Bacterial Reverse Mutation Test

The results of the reverse mutation test are shown in Table 2. As shown in this table, the positive controls (with or without S9) induced revertant colonies at a rate higher than twofold that of the untreated control group and were significantly different from the untreated control group (*P< 0.01*), implying mutagenic effects. For the Suo Yang dose groups, no concentration induced more than double the number of revertant colonies. Furthermore, no significant differences were observed between the Suo Yang dose groups and the untreated control group (*P> 0.05*). Thus, the bacterial reverse mutation test for Suo Yang was concluded to be negative independently of the effects of the liver microsomal enzyme system.

#### 3.2.2. Mouse Bone Marrow Micronucleus Assay

Table 3 shows the results of the bone marrow micronucleus test in mice. Micronuclei were observed, and the micronuclei rate was calculated among 2000 PCEs in all groups. No significant differences in the ratio of micronuclei relative to the total number of PCEs were observed in the Suo Yang-treated groups compared with the solvent control group (*P>0.05*). By contrast, the positive control group had a significantly higher ratio than the solvent control group (*P*< 0.01), indicating that the tested mice are sensitive and provided reliable results. In the prescribed dose ranges, Suo Yang showed negative results in the mouse bone marrow micronucleus assay. These results indicated that Suo Yang did not result in mutagenesis.

#### 3.2.3. Spermatocyte Chromosome Aberration Assay in Mice

Table 4 shows the results of the spermatocyte chromosome aberration assay. After administration of different Suo Yang doses for 5 days, the frequencies of chromosome abnormalities and abnormal cell rates in all dose-treated groups showed no significant difference compared with those in the solvent control group (*P > 0.05*). However, the positive control group showed significant differences in both the frequency of chromosome abnormalities and abnormal cell rate compared with the solvent group (*P<0.05*). The results of the test suggested that Suo Yang does not cause spermatocyte chromosomal aberrations in mice.

### 3.3. 90-Day Repeated Oral Toxicity Study

#### 3.3.1. General Conditions, Body Weight, and Food Utilisation Rate

**Figure 1:**
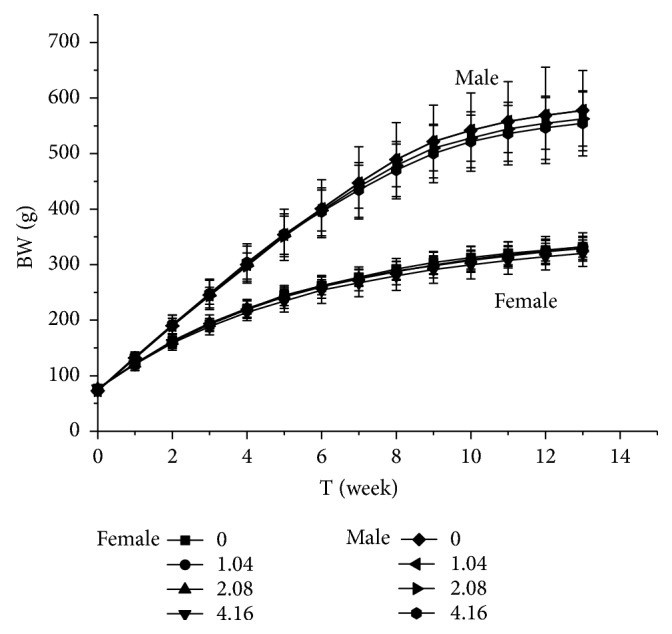
*Food utilisation rate in rats*. The following doses of Suo Yang were administered to the four groups: control group, 0 g/kg; low dose, 1.04 g/kg; medium dose, 2.08 g/kg; and high dose, 4.16 g/kg. The values are expressed as the means ± SD (n = 10 rats for 90 days). *∗ P* < 0.05; *∗∗ P*< 0.01 statistically significant compared to the control group.

**Figure 2:**
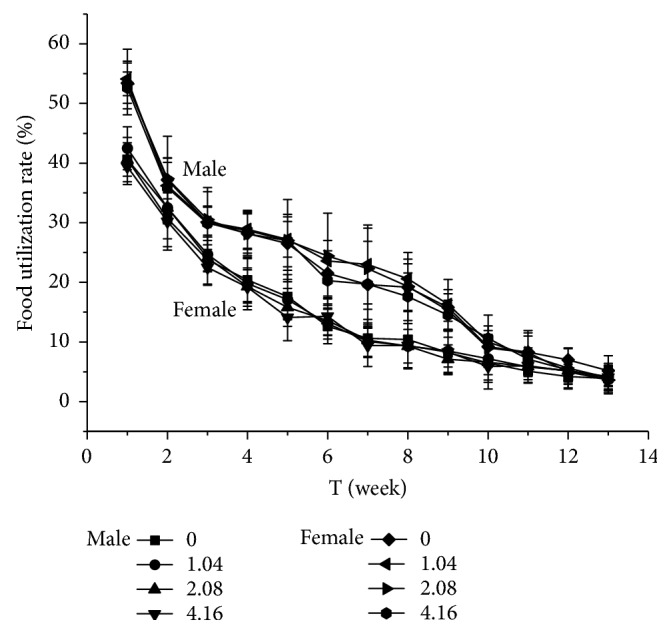
*Body weight in rats*. The following doses of Suo Yang were administered to the four groups: control group, 0 g/kg; low dose, 1.04 g/kg; medium dose, 2.08 g/kg; and high dose, 4.16 g/kg. The values are expressed as the means ± SD (n = 10 rats for 90 days). *∗ P* < 0.05; *∗∗ P*< 0.01 statistically significant compared to the control group.

During the oral administration of Suo Yang and the vehicle for 90 days, all rats were in good condition. No significant abnormalities in fur colour, behaviour, food consumption, or drinking were observed between the dose groups and the control group in both male and female rats. The results for body weight and food utilisation rate are shown in Figures 1 and 2. Body weight and food utilisation rate of all dose-treated groups were not significantly different from those of the control group (*P* > 0.05). This finding indicated that Suo Yang did not have any significant harmful effects on body weight and food utilisation.

#### 3.3.2. Haematological and Clinical Biochemical Analysis

The results of haematological biochemistry analysis are shown in Table 5. After statistical analysis, all of these markers, including AST, ALT, TP, ALB, TG, TC, BUN, CREA, and GLU, showed no significant differences in the treatment group compared with the control group in both female and male rats (*P* > 0.05). The results of the clinical biochemical biochemistry parameters are shown in Table 6. The level of Eos was slightly higher in female rats in all dose groups than in females in the control group. However, these differences were neither significant nor dose dependent. No significant differences were observed in any other parameters between the control group and any of the dose groups (*P* > 0.05).

#### 3.3.3. Organ Weight, Relative Organ Weight, and Histopathological Analysis

The organ weights and relative organ weights of rats are shown in Table 7. All the examined organs showed no differences between the control group and dose groups. In addition, no significant differences were found in any organ relative weights between the control group and dose groups in both male and female rats (*P *> 0.05).

**Figure 3:**
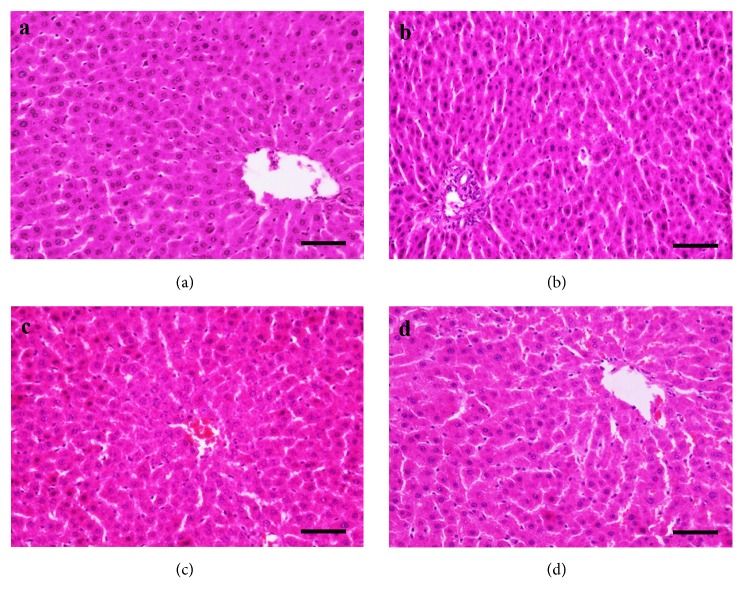
*H&E staining of the liver*. (a) Female control group, (b) female high dose group, (c) male control group, and (d) male high dose group; n = 10; scale bar = 50 *μ*m; magnification, 400×.

**Figure 4:**
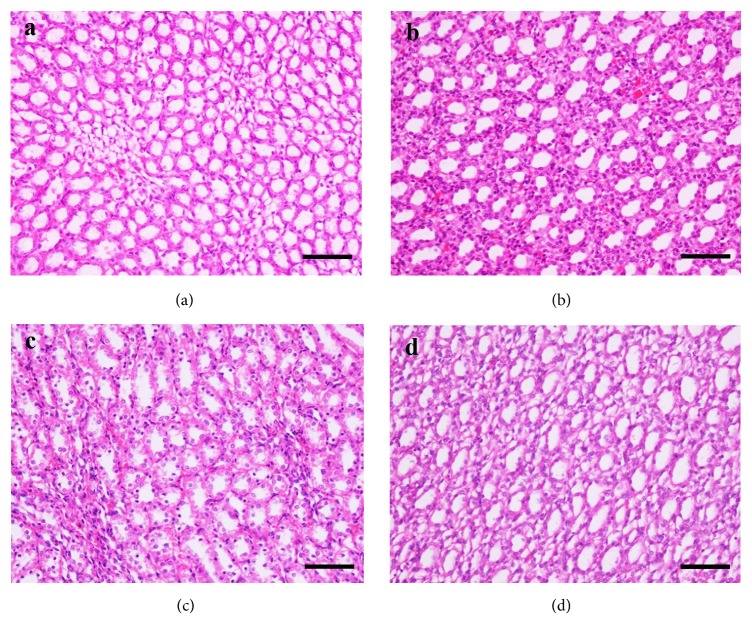
*H&E staining of the kidney*. (a) Female control group, (b) female high dose group, (c) male control group, and (d) male high dose group; n = 10; scale bar = 50 *μ*m; magnification, 400×.

The results of pathological examinations are shown in Figures 3 and 4. In general, no remarkable gross lesions were observed in the liver or kidney of rats in the dose group or the control group. However, microscopic examination showed minor differences in the liver. For several rats, slight fatty degeneration of hepatocytes in the liver was observed in the solvent control group.

## 4. Discussion

With the increasing use of complementary and alternative medicine, herbal medicine has become increasingly popular [15]. Traditional medicine has been employed for preventing and treating various diseases for hundreds of years; in addition, it has become a popular alternative choice to conventional medicine [16]. Although many herbal medicine interventions are available, including some that have been verified by clinical trials, the efficacy and safety of most remain unclear [17, 18]. Suo Yang is one of the most popular herbal medicines in China, and many studies have reported its pharmacological efficacies and benefits, such as antiageing [19], antioxidant, and free radical scavenging activities [20] and enhancement of fertility [21]. However, little information exists about its risk and safety, such as its acute, genetic and subchronic toxicity. The safety of a medicine may be the most important concern [22]. To establish evidence-based toxicity data for Suo Yang, we performed this research.

The acute toxicity of Suo Yang was investigated using in vivo experiments. No abnormalities or mortality was found in mice treated with Suo Yang at a dose of 18 g/kg, equivalent to 432 times the clinical human dosage. In addition, the approximate lethal doses of Suo Yang for male and female animals were found to be higher than 15 g/kg. Therefore, Suo Yang could be regarded as a nontoxic herbal medicine.

The genotoxicity of the Suo Yang was investigated using in vitro and in vivo assays. In the Ames test (bacterial reverse mutation test), no significant differences were observed between the Suo Yang dose groups and the untreated group (*P> 0.05*). We could conclude that Suo Yang did not exhibit mutagenetic activity in the presence or absence of metabolic activation with S9 mix. The Ames mutagenicity assay is a short-term bacterial reverse mutation assay specifically designed to detect a wide range of chemical substances that can result in genetic damage and lead to gene mutations [23]. The results of this assay in the present study were similar to those of a previous study showing that Suo Yang can protect against menadione cytotoxicity in cells [19]. In the mouse bone marrow micronucleus assay, the Suo Yang dose group did not show an increase in the micronuclei rate compared with the negative control group (*P> 0.05*). Suo Yang did not damage chromosomes or the mitotic apparatus when orally administered to mice at different doses. This assay is considered a preferable method for evaluating chromosome damage because it allows the determination of both chromosomal loss and breakage [24, 25]. In the spermatocyte chromosome aberration assay, in comparison with the control group, Suo Yang dose groups showed no significant difference in chromosomal abnormality rate (*P> 0.05*). This result suggested that Suo Yang did not show teratogenicity in the mice. Moreover, this result is consistent with a previous study, which suggested that extracts of Suo Yang can enhance sperm motility [21]. Based on the above three genotoxicity tests, Suo Yang can be concluded to not induce genotoxicity.

In the 90-day repeated oral toxicity assay, the food utilisation rate and body weight showed no significant difference between the control group and dose groups in both male and female rats (*P> 0.05*). In terms of haematological and clinical biochemical parameters, no significant differences were observed between the control and dose groups in either female or male rats (*P* > 0.05). For all dose groups, the Eos level was slightly higher than that of the control group. However, because these changes showed no statistically significant difference (*P> 0.05*) and were within the normal physiological ranges, they were not considered to be related to Suo Yang toxicity [26]. For all examined organs (including the liver, kidneys, spleen, and testes), weight and relative weight showed no significant differences between the dose groups and the control group (*P* > 0.05). In the histopathological examinations, slight fatty degeneration of hepatocytes in the liver was observed in the solvent control and high dose groups. However, these lesions were minimal and rare, so they were considered incidental, and this effect can be reasonably considered nontoxicological. Overall, these results revealed that Suo Yang had no toxic effect under our experimental conditions.

## 5. Conclusion

Single-dose acute toxicity, genotoxicity, and 90-day repeated oral toxicity of Suo Yang were examined to identify toxicity and to ensure pharmaceutical safety. As a result, no toxicity or mutagenicity was observed in all studies, including acute toxicity, Ames test, mouse bone marrow micronucleus assay, and 90-day repeated oral toxicity. In conclusion, the toxicity studies indicated that at the tested doses, Suo Yang can be regarded as a safe and nontoxic pharmaceutical material. Therefore, further investigation of the active functions of Suo Yang as a safe herbal medicine is worthwhile.

## Figures and Tables

**Table 1:** The results of the acute toxicity test in mice.

Sex	Dose (g/kg)	Changes in weight		NO. death	MTD (g/kg)
		Initial weight (g)	Final weight (g)		
Male	18	19.8 ± 1.1	39.2 ± 0.7	0	>15
Female	18	20.0 ± 1.1	34.8 ± 1.0	0	>15

Values are expressed as the means ± D (n = 10).

**Table 2:** The results of the bacterial reverse mutation test.

Chemical	Dose (*μ*g/plate)		Revertant colonies/plate				
		S9	TA97	TA98	TAl00	TAl02	TA1535
Suo Yang	8	-	113 ± 9	37 ± 8	172 ± 21	286 ± 10	16 ± 2
	40	-	120 ± 13	40 ± 1	172 ± 19	289 ± 13	10 ± 4
	200	-	119 ± 15	46 ± 4	136 ± 12	262 ± 10	10 ± 8
	1000	-	125 ± 17	46 ± 2	134 ± 5	276 ± 7	13 ± 4
	5000	-	121 ± 15	39 ± 6	151 ± 29	256 ± 5	8 ± 3
Untreated control	0	-	118 ± 14	37 ± 7	135 ± 18	275 ± 19	10 ± 3
Solvent control 1	0	-	111 ± 5	38 ± 8	151 ± 14	292 ± 3	11 ± 6
Solvent control 2	0	-	121 ± 10	41 ± 4	143 ± 15	288 ± 14	11 ± 5
Positive control 1	50	-	1723 ± 35^*∗∗*^	818 ± 67^*∗∗*^		786 ± 56^*∗∗*^	
Positive control 2	1.5	-			1016 ± 90^*∗∗*^		462 ± 23^*∗∗*^
Suo Yang	8	+	121 ± 7	40 ± 1	167 ± 25	277 ± 22	15 ± 3
	40	+	114 ± 11	37 ± 10	151 ± 35	272 ± 10	9 ± 4
	200	+	127 ± 14	41 ± 5	158 ± 34	273 ± 21	16 ± 2
	1000	+	123 ± 12	39 ± 7	165 ± 34	263 ± 19	13 ± 5
	5000	+	120 ± 8	33 ± 4	154 ± 27	277 ± 23	13 ± 7
Negative control	0	+	126 ± 5	38 ± 10	150 ± 13	262 ± 13	13 ± 3
Solvent control 1	0	+	124 ± 19	36 ± 6	177 ± 5	279 ± 14	11 ± 6
Solvent control 2	0	+	122 ± 6	37 ± 6	148 ± 14	287 ± 7	11 ± 6
Positive control 3	10	+	1533 ± 90	2141 ± 207	921 ± 47		
Positive control 4	50	+				796 ± 84^*∗∗*^	
Positive control 5	200	+					518 ± 19^*∗∗*^

Values are expressed as the means ± SD (n = 10).

Solvent control 1: sterile water; Solvent control 2: DMSO; Positive control 1: dimethylaminobenzene diazosulfonate sodium; Positive control 2: sodium azide; Positive control 3: 2-aminoanthracene; Positive control 4: 1,8-dihydroxyanthraquinone; Positive control 5: Endoxan.

*∗∗P*< 0.01 statistically significant compared to the untreated control group.

**Table 3:** The results of bone marrow cell micronuclei in mice.

Sex	Dose (g/kg)	Cells	Micronuclei	Micronuclei Rate (%)
Female	0	5 × 2000	21	2.1 ± 0.7
	2.25	5 × 2000	19	1.9 ± 0.8
	4.5	5 × 2000	24	2.4 ± 0.4
	9	5 × 2000	19	1.9 ± 0.4
	Endoxan	5 × 2000	105	10.5 ± 2.2^*∗*^

Male	0	5 × 2000	23	2.3 ± 0.6
	2.25	5 × 2000	20	2.0 ± 0.9
	4.5	5 × 2000	18	1.8 ± 1.1
	9	5 × 2000	18	1.8 ± 0.7
	Endoxan	5 × 2000	120	12.0 ± 2.4^*∗*^

Values are expressed as the means ± SD (n = 10).

0: the negative control, Endoxan: the positive control; ^*∗*^*P*<0.05 compared with the negative control.

**Table 4:** The results show spermatocyte chromosomal aberrations in mice.

			Types of chromosomal dysgenesis				
Dose	No. of cells	Fragment	Microchromosome	Translocation	Chromosomal abnormality rate (%)	Abnormal cells	Abnormal cell rate (%)
0	500	2	0	0	0.4	2	0.4
2.25	500	2	0	0	0.4	2	0.4
4.5	500	1	0	0	0.2	1	0.2
9	500	2	0	0	0.4	2	0.4
Endoxan	500	5	0	7	2.40^*∗*^	11	2.20^*∗*^

n = 5.

0: the negative control; Endoxan: the positive control; ^*∗*^p<0.05 compared with the negative control group.

**Table 5:** The results of haematological biochemistry parameters in rats.

	Parameters	Dose (g/kg BW)			
		0	1.04	2.08	4.16
Female	AST (U/L)	157 ± 7	157 ± 7	156 ± 8	158 ± 7
	ALT (U/L)	35 ± 2	35 ± 2	35 ± 3	35 ± 3
	TP (g/L)	60.3 ± 2.8	59.6 ± 2.5	60.4 ± 2.4	60.4 ± 2.2
	ALB (g/L)	30.4 ± 1.9	30.6 ± 1.7	30.6 ± 1.7	31.0 ± 1.7
	TG (mmol/L)	0.54 ± 0.09	0.56 ± 0.11	0.54 ± 0.09	0.56 ± 0.08
	TC (mmol/L)	1.92 ± 0.20	1.82 ± 0.18	1.84 ± 0.15	1.87 ± 0.15
	BUN (mmol/L)	5.5 ± 0.8	5.2 ± 0.9	5.3 ± 0.8	5.4 ± 0.8
	CREA (*μ*mol/L)	49 ± 4	49 ± 5	47 ± 5	48 ± 4
	GLU (mmol/L)	6.31 ± 0.53	6.35 ± 0.63	6.50 ± 0.55	6.66 ± 0.50

Male	AST (U/L)	168 ± 9	164 ± 9	167 ± 8	167 ± 7
	ALT (U/L)	36 ± 3	36 ± 3	37 ± 2	37 ± 3
	TP (g/L)	60.7 ± 3.0	60.9 ± 3.1	61.2 ± 3.5	61.6 ± 3.2
	ALB (g/L)	30.4 ± 2.1	30.5 ± 2.3	30.0 ± 2.2	30.3 ± 2.5
	TG (mmol/L)	0.61 ± 0.11	0.65 ± 0.11	0.62 ± 0.12	0.6 ± 0.1
	TC (mmol/L)	1.87 ± 0.19	1.90 ± 0.23	1.89 ± 0.17	1.92 ± 0.16
	BUN (mmol/L)	4.9 ± 1.0	5.0 ± 1.0	5.0 ± 1.1	5.1 ± 1.0
	CREA (*μ*mol/L)	47 ± 5	48 ± 4	49 ± 5	48 ± 5
	GLU (mmol/L)	6.66 ± 0.62	6.659 ± 0.63	6.673 ± 0.62	6.72 ± 0.56

Values are expressed as the means ± SD (n = 10).

**Table 6:** The results of clinical biochemical parameters in rats.

	Parameters	Dose (g/kg BW)			
		0.00	1.04	2.08	4.16
Female	RBC	6.7 ± 0.5	6.9 ± 0.4	6.9 ± 0.4	6.6 ± 0.5
	HGB (g/L)	139 ± 7	142 ± 6	136 ± 5	140 ± 5
	PLT	650 ± 80	629 ± 80	617 ± 97	640 ± 86
	WBC	4.9 ± 0.5	4.7 ± 0.5	4.6 ± 0.5	4.9 ± 0.4
	Neu (%)	18.8 ± 5.5	17.9 ± 4.6	18.1 ± 5.8	21.5 ± 4.5
	Lym (%)	75.2 ± 5.6	75.2 ± 4.9	75.0 ± 6.0	72.0 ± 4.9
	Mon (%)	4.3 ± 0.7	4.8 ± 0.9	4.9 ± 0.8	4.6 ± 1.0
	Eos (%)	1.1 ± 0.5	1.5 ± 0.5	1.4 ± 0.5	1.4 ± 0.5
	Bas (%)	0.7 ± 0.2	0.6 ± 0.2	0.6 ± 0.2	0.7 ± 0.2

Male	RBC	6.9 ± 0.4	6.9 ± 0.4	6.9 ± 0.4	6.7 ± 0.5
	HGB (g/L)	141 ± 7	139 ± 7	139 ± 6	142 ± 7
	PLT	642 ± 70	632 ± 98	630 ± 77	594 ± 86
	WBC	4.9 ± 0.5	4.7 ± 0.5	4.6 ± 0.5	4.9 ± 0.4
	Neu (%)	18.8 ± 5.5	17.9 ± 4.6	18.1 ± 5.8	21.5 ± 4.5
	Lym (%)	73.1 ± 5.5	73.6 ± 5.3	73.8 ± 5.2	71.9 ± 4.7
	Mon (%)	4.6 ± 0.9	4.6 ± 0.8	4.9 ± 1.1	5.1 ± 0.8
	Eos (%)	1.4 ± 0.6	1.5 ± 0.5	1.4 ± 0.4	1.4 ± 0.4
	Bas (%)	0.6 ± 0.2	0.6 ± 0.2	0.6 ± 0.2	0.6 ± 0.2

Values are expressed as the means ± SD (n = 10).

**Table 7:** The results of relative organ weights in rats.

	Parameters		Dose (g/kg)			
			0	1.04	2.08	4.16
Female	Body weight		306.9 ± 17.6	306.7 ± 24.2	304.4 ± 21.7	295.3 ± 22.3
	Relative/%	Liver	2.51 ± 0.21	2.49 ± 0.18	2.50 ± 0.16	2.53 ± 0.22
		Kidney	0.65 ± 0.05	0.63 ± 0.05	0.62 ± 0.07	0.64 ± 0.06
		Spleen	0.16 ± 0.03	0.15 ± 0.03	0.16 ± 0.02	0.17 ± 0.03

Male	Body weight		503.5 ± 47.7	504.8 ± 71.2	489.2 ± 48.2	481.2 ± 59.7
	Relative/%	Liver	2.77 ± 0.32	2.72 ± 0.22	2.73 ± 0.32	2.82 ± 0.51
		Kidney	0.67 ± 0.03	0.63 ± 0.05	0.66 ± 0.07	0.65 ± 0.04
		Spleen	0.16 ± 0.03	0.16 ± 0.03	0.16 ± 0.01	0.17 ± 0.03
		Testis	0.67 ± 0.06	0.66 ± 0.08	0.67 ± 0.08	0.70 ± 0.08

Values are expressed as the means ± SD (n = 10).

## Data Availability

All data generated or analysed for this study are included in this published article. Raw data are available from the corresponding author on reasonable request.

## Abbreviations

RBC: Red blood cells
HGB: Haemoglobin
PLT: Platelets
WBC: White blood cells
Neu: Neutrophil
Lym: Lymphocyte
Mon: Monocytes
Eos: Eosinophils
Bas: Basophils
AST: Aspartate aminotransferase
ALT: Alanine aminotransferase
TP: Total protein
ALB: Albumin
TG: Total triglycerides
TC: Total cholesterol
BUN: Blood urea nitrogen
CREA: Creatinine
GLU: Glucose.
